# Does Work Affect Personality? A Study in Horses

**DOI:** 10.1371/journal.pone.0014659

**Published:** 2011-02-09

**Authors:** Martine Hausberger, Christine Muller, Christophe Lunel

**Affiliations:** Université de Rennes 1, UMR 6552 Ethologie Animale et Humaine, Unité Mixte de Recherche CNRS, Campus de Beaulieu, Rennes, France; Georgia State University, United States of America

## Abstract

It has been repeatedly hypothesized that job characteristics are related to changes in personality in humans, but often personality models still omit effects of life experience. Demonstrating reciprocal relationships between personality and work remains a challenge though, as in humans, many other influential factors may interfere. This study investigates this relationship by comparing the emotional reactivity of horses that differed only by their type of work. Horses are remarkable animal models to investigate this question as they share with humans working activities and their potential difficulties, such as “interpersonal” conflicts or “suppressed emotions”. An earlier study showed that different types of work could be associated with different chronic behavioural disorders. Here, we hypothesised that type of work would affect horses' personality. Therefore over one hundred adult horses, differing only by their work characteristics were presented standardised behavioural tests. Subjects lived under the same conditions (same housing, same food), were of the same sex (geldings), and mostly one of two breeds, and had not been genetically selected for their current type of work. This is to our knowledge the first time that a direct relationship between type of work and personality traits has been investigated. Our results show that horses from different types of work differ not as much in their overall emotional levels as in the ways they express emotions (i.e. behavioural profile). Extremes were dressage horses, which presented the highest excitation components, and voltige horses, which were the quietest. The horses' type of work was decided by the stall managers, mostly on their jumping abilities, but unconscious choice based on individual behavioural characteristics cannot be totally excluded. Further research would require manipulating type of work. Our results nevertheless agree with reports on humans and suggest that more attention should be given to work characteristics when evaluating personalities.

## Introduction

Despite early findings showing that job characteristics are related to changes in personality in humans, revealing reciprocal relationships between personality and work [1], more recent models of personality, such as the five-factor model [2], still omit effects of life experience on personality traits.

Nevertheless work stressors are often associated with impaired psychological functioning, such as increased anxiety that can persist beyond the work situation [3]. According to Roberts et al. [4], work experiences have the potential to modify basic personality dispositions. Work satisfaction leads to increased levels of emotional stability [5].

Amongst the predominant work stressors that can have consequences outside the work situation are interpersonal stressors [6] and emotion management (suppressed expressions of emotion at work) [7]. Horses have in common with humans a working activity where “interpersonal” (horse/human, horse/horse) conflicts may arise and where emotions may have to be suppressed (e.g. [8]).

A recent study revealed that the type of work for which a horse was used could lead to chronic behavioural disorders outside the work situation [9]. Thus, dressage horses, trained to obey to precise orders (i.e. to suppress any kind of emotional expression), presented stereotypic behaviours more frequently and “stronger” forms of stereotypies than did horses from other disciplines. Clearly, if work characteristics can induce chronic behavioural problems in horses, it could also affect their personality, as it does in humans [4].

Studies of horses' personality use behavioural tests (e.g. [10]-[12]), questionnaires (e.g. [13]-[14]), or both (e.g. [15]-[18]). All these studies converge to show high levels of individual variations in traits like fearfulness or gregariousness. Factors, like paternal origin (e.g. [10]), breed ([19]-[20]) or environmental conditions (e.g. [21]), can influence temperament / personality traits.

Hausberger et al.'s [19] study of over 700 horses revealed that several genetic and environmental factors, including type of work, were involved in explaining horses' personality traits. Dressage horses reacted the most strongly to emotionality tests. However, whether work or associated characteristics (way of life, genetic origin) were involved could not be clearly determined.

Here, we hypothesised that type of work would affect horses' personality. Therefore over one hundred adult horses, differing only by their work characteristics were presented standardised behavioural tests. Subjects lived under the same conditions (same housing, same food), were of the same sex (geldings), and mostly one of two breeds, and had not been genetically selected for their current type of work.

This is to our knowledge the first time that a direct relationship between type of work and personality traits has been investigated. Our results show that horses from different types of work differ not as much in their overall emotional levels as in the ways they express emotions (i.e. behavioural profile). Extremes were dressage horses, which presented the highest excitation components, and voltige horses, which were the quietest.

## Materials and Methods

Experiments complied with the current French laws (Centre National de la Recherche Scientifique) related to animal experimentation and were in accordance to the European directive 86/609/CEE. Only behavioural observations were performed. The riding school staff was responsible for all animal husbandry and care, as this experiment involved horses from the “field” (no laboratory animals).

### The subjects

One hundred and nineteen horses were tested at the “Ecole Nationale d'Equitation” at Saumur (France) between October and December 1994. They were 4–20 year-old geldings and were all housed under the same conditions, in single boxes, and ridden for one hour every day. They were fed pellets four times a day and hay twice a day and had water at libitum. Pellets were distributed through automatic feeders, and diets or quantities of food did not differ according to type of work.

The horses belonged to two breeds: French Saddlebreds (N = 89) and Angloarabs (N = 30).

The horses were divided into six groups according to type of work (see description in Appendix S1): eventing (n = 11), show jumping (n = 41), advanced riding school (n = 18), dressage (n = 29), high school (n = 15) and voltige (n = 5) (Table 1). These groups differed only by type of work as we ensured that 1) age ranges did not differ among groups (eventing: 7.82±1.94; show jumping: 9.1±3.3; advanced riding school: 10.6±3.29; dressage: 9.34±3.29; high school: 10.8±4; voltige: 12.8±4.44 years old); 2) their diets were the same: commercial pellets were provided by automatic feeders (general to the whole facility) to all horses, at the same feeding times, 4 times a day (6.30 am, 11.30 am, 4.15 pm, 6.30 pm), the quantities were determined only by size / weight, not by type of work; 3) all subjects had been working in their type of work for at least 1 year; 4) all had arrived at this riding school when they were 4 to 5 years old; 5) horses of all types of work were mixed in different locations over the facility. Apart from the five voltige horses, none of these horses had been selected for a particular type of work when they had arrived. French saddlebreds were only selected for jumping and therefore there were no bloodlines selected for dressage, for example, in our sample that could explain the behavioural differences observed (see results). All horses received basic training, especially for jumping. Selection was based mainly on jumping ability, the best jumpers (free jump in particular) were allocated to jumping and eventing, then, according to the quality of their paces and conformation the remaining horses were allocated either to dressage or to high school. Advanced riding school horses tended to be less competitive overall. Information on possible changes during the horses' careers was lacking, but stability in type of work prevailed once the initial choice was made. Therefore, time spent doing their current type of work was probably related to the subject's age. Since we found no relationship between age and behaviour or emotionality index (p>0.05 in all cases), one year in their current work seems sufficient to influence personality. Moreover, several bloodlines were found for different types of work.

**Table 1:** Distribution of horses in relation to type of work.

	Jumping	Eventing	Instruction	Voltige	Dressage	High School	N
**N**	41	11	18	5	29	15	119
**AA**	6	5	11	0	7	1	30
**SF**	35	6	7	5	22	14	89

AA = Angloarabs; SF = French Saddlebreds; N = total number.

In fact, 98 different sires were involved, but 8 well known jumping stallions had more than one offspring in our sample (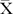 = 1.21±0.58 offspring per stallion). The half siblings (same sire, different mother) of 23 of the 36 horses concerned were doing different types of work (high school/show jumping, eventing/dressage, eventing/jumping, jumping/dressage, voltige/dressage) indicating that paternal origin was not determinant in the distribution of horses among types of work. Moreover, 22 of the 98 sires had been race horses and 27 show jumping horses, the remaining sires had been selected early on morphological characteristics (and had not worked) in order to promote bloodlines for racing or show jumping (N = 15). No relationships between type of work of our subjects and that of their sire could be evidenced.

### Experimental tests

Our experimental tests are commonly used to assess emotionality and learning abilities (review in [22]). Moreover, results of these tests correlated with estimates of personality traits by users in working situations [16].

#### Tests estimating emotional reactions

Three tests, described by Wolff et al. [10] were used:

“Arena test”: horses were released alone in a familiar arena (where they are ridden) and their behaviour was scan recorded every 10 s for 10 min (see also [23], [24]. This differs from classical open-field tests, as the site is familiar and this test has been shown to estimate the effects of social separation [16].
“Novel object” test: an object was placed in the arena and a horse was released for 5 min: its behaviour, locomotion, gazes and approaches were recorded. Correlations have been found between reactions to this test and estimations of nervousness by users [16].
“Bridge” test: a horse was led using a halter over an unknown obstacle built with a foam mattress [19] (planks: [10]; concrete blocks: [11]). Reactions to this test have been shown to correlate with evaluations of fear by horse users [16]. Visser et al. [25] correlated standing in front of the bridge (refusal to cross) with “spooky”.

The horses had worked the day before a test. The tests were carried out before the horses worked that day. Each horse was presented each test only once to avoid habituation [26], [27].

### General procedure for the “arena” and “novel object” tests

The tests took place in the usual working (30×15 m) arenas, with the ground covered with sand. The subject was released as soon as it came into the arena and the observer, remaining immobile in a corner of the arena far from the entrance, recorded its behaviour with a voice cassette recorder, using the “instantaneous scan sampling” and “all occurrences” methods (Altmann [28]). Our procedure was the same as Wolff et al.'s [10]: we observed the horses for either 10 min (arena test) or 5 min (novel object test) after they had been released. Scans were recorded every 10 s, yielding respectively 60 and 30 data per horse for each test.

An object, unknown to the horses, was designed for the novel object test (see also [17]). Six metal rails (square section: 9 cm) formed a cage (100×80×80 cm) onto which one long red fluorescent ribbon was attached, crossing the rails on each side. This object was placed to the side of the entrance so that the horse could see it only after it had entered the arena. A 10 m diameter circle was drawn on the sand around the object to help estimate distance between horse and object (±5 m from the object).

#### Observation procedure

Rare or brief behavioural patterns were recorded every time they occurred: snorts, pawing, defecation, rolling, whinnying (“all occurrences method”, [28]).

The behavioural patterns recorded using scan sampling were: (1) standing; (2) exploration: the horse walks slowly with its neck held horizontally or lowered, ready to stop and to sniff the ground or a wall. This is the characteristic slow walk of a quiet horse in a calm situation; (3) sustained walk: the horse walks energetically and looks ahead or around; (4) trot: a two-beat gait; (5) passage: an animated form of trot when the legs are raised higher; (6) canter: a three-beat gait; (7) vigilance: the horse stands still and holds its neck high, with intently oriented head and ears; (8) tail: hangs down or is raised, the fleshy portion of the tail is then almost or completely upright and the long tail hairs make a showy display [29].

#### Data analyses (see [10])

Two types of analyses were used. Frequencies of occurrence of behavioural patterns were calculated and compared among individuals. An index, used in previous studies (e.g. [19]) and based on both behavioural patterns and their frequencies of occurrence, « ranked » reactivity of horses in each situation. Values were attributed to the behavioural patterns according to their degree of specificity and corresponding level of arousal (see [30]). These values were: exploration (slow walk) = 1, sustained walk = 2, trot or canter = 3, vigilance = 4, whinnying = 5, passage, snorting or tail raised = 6. These values were multiplied by the number of times the corresponding pattern was observed. Remember, these values only give a rank indication and do not represent real data. Thus an animal with an index twice as high as that of another horse is not necessarily twice as reactive.

### General procedure for the bridge test

A foam mattress (200×100×10 cm), covered with a brown and white check oilcloth (squares: 2×2 cm), was called the bridge. The starting line was drawn on the sand 2 m in front of the bridge. The experimenter (C. Muller) led the horse using a halter with a rope attached to the ring and tried to make it cross the bridge. She was not allowed to touch or to talk to the horse. Her activity was limited to pulling slightly on the rope if necessary. All tests were made by the same person who was not familiar to the test horses.

Many animals avoided walking on the bridge and passed by on one side. In this case, they were led back to the starting line and a new trial began (the stopwatch was stopped until the new start). The test was stopped either when the horse had crossed the bridge placed at least three feet, or after 10 min, the maximum allowed for this test.

Data recorded were the total time required to cross the bridge.

### Statistical analyses

Two statistical approaches were used: a Factorial Correspondence Analysis (FCA) and non-parametric statistical tests.

Factorial analysis is a descriptive but very informative approach yielding a simultaneous plot of both groups of variables tested (here emotional reactions and horses characterised by their type of work) and a visualisation of their relationship.

Non-parametric statistical tests were used, as normality of data was not ensured: χ^2^ tests compared the numbers of animals performing given behavioural patterns between groups. Mann-Whitney and Kruskal-Wallis non-parametric tests compared frequencies of behaviours between groups. Spearman correlation tests compared rank orders.

## Results

### General behaviour

In both the arena and novel object tests the main behavioural patterns observed were mostly standing (70.6±28.3 %, 54.8±39.3 % respectively), exploring (9.4±11.2 %, 12.1±16 %) or sustained walk (8.5±10.46 %, 10.9±14.7 %), but rarely canter (4.4±7.3 %, 3.8±5.3 %) or passage (0.4±1.3 %, 0.4±1.2 %). Defecation (0.04±0.2 %) was rarely observed. In all cases important interindividual variations were observed as revealed by high coefficients of variation (e.g. 397 for passage in the arena test) and emotionality indices ranged from 0 to 294 (56.06±59.93 %). Pawing and emotionality index were correlated positively (N = 20, r_s_ = 0.389, p = 0.0024, r_s_ = 0.258 p = 0.0049 for each test respectively).

In the novel object test, distances between horse and object were correlated with emotionality index: the longer a horse spent at less than 5 meters from the object, the lower its emotionality index (r_s_ = 0.287, p = 0.0035); conversely, the longer a horse spent away from the object, the higher its emotionality index (r_s_ = 0.212, p = 0.0089). Individual emotionality Indices were correlated between arena and novel object tests (r_s_ = 0.482, p = 0.0001).

In the bridge test, most horses (63 %) crossed the bridge in the allocated time (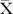 = 101±94 s). However, large individual differences were observed in the time required (7 to 392 s). Angloarab horses took longer to cross the bridge (368.88±238.63 s) than did French Saddlebreds (258.39±254.07 s) (Mann Whitney U test N1 = 32, N2 = 88, U = 1043.5 p = 0.026).

### Reactions to tests according to type of work

No differences were found between breeds for either the arena or the novel object tests for any behavioural pattern, therefore data were pooled for these tests. Although type of work did not influence significantly emotionality indices in the arena test (Kruskal Wallis test: H = 8, p = 0.15), its influence approached statistical significance in the novel object test (H = 3.41, p = 0.06) (Figure S1). Differences in behavioural profiles according to type of work appeared clearly in the behavioural profiles expressed during both tests (Figures S2 and S3).

The first two axes of the FCA on occurrences of behavioural patterns in the novel object test (excluding voltige horses, as they had been selected for temperament), accounted for 48 % of the variance (Figure S2, Table 2). Axis 1 segregated “quieter” behaviour (touching the object) from excited behaviour (vigilance, snorting, tail raised, cantering), whereas axis 2 segregated gazes and contact with novel object from excited behaviours like tail raised. Jumping horses were more prone to touch the object, while high school and dressage horses showed more high locomotor and excited behavioural patterns, such as snorting, tail raised or vigilance. The same general pattern was observed for arena test data: the first two axes accounted for 50 % of the variance (Figure S3, Table 3)

**Table 2:** Factor loadings of the Factorial Correspondence Analysis (FCA) on the frequencies of behavioural patterns in the novel object test.

	Factor loadings of variables			
		F1	F2	F3
**Behavioural pattern**	TrotPassage	358	8	24
	Canter	307	31	2
	Vigilance	356	39	5
	Walk	82	69	22
	Slow Walk	30	181	264
	Tail raised	691	174	14
	Rolling	4	6	472
	Pawing	15	18	568
	Snorting	285	33	0
	Touch	541	422	17
	Gaze	37	598	205
**Type of work**	Eventing	21	51	55
	Jumping	775	66	19
	Dressage	411	225	82
	High-school	737	106	12
	Advanced riding school	367	362	87

Factor loadings are the squared correlation coefficients between the variables and factors.

**Table 3:** Factor loadings of the Factorial Correspondence Analysis (FCA) on the frequency of behavioural patterns in the arena test.

	Factor loadings of variables			
		F1	F2	F3
**Behavioural pattern**	Trot	27	98	150
	Canter	125	495	16
	Vigilance	104	733	22
	Passage	72	0	375
	Tail raised	425	17	107
	Rolling	638	1	218
	Pawing	665	12	132
	Snorting	90	19	9
**Type of work**	Eventing	31	317	144
	Jumping	845	115	5
	Dressage	828	17	0
	High-school	662	74	5
	Advanced riding school	211	3	70

Factor loadings are the squared correlation coefficients between the variables and factors.

These profiles were confirmed when behavioural patterns were compared in detail one by one. Thus, in the arena test, the number of horses performing passage (df = 5, χ^2^ = 13.11, p = 0.02; without voltige: dfl = 4, p = 0.017) or rolling (χ^2^ = 11.05, p = 0.05) differed according to the type of work: more dressage ((χ^2^ = 3.32, p = 0.06) and high school horses (df = 1, χ^2^ = 4.37, p = 0.037) but fewer jumping horses (df = 1, χ^2^ = 5.09, p<0.05) performed passage; less high school horses rolled (χ^2^ = 3.84, p = 0.05), and when taken into consideration, voltige horses rolled more (χ^2^ = 6.8, p = 0.009).

The occurrence of passage also differed clearly according to the type of work (H = 13.41, df = 5, p = 0.02; H = 12.1, df = 4, p = 0.017 without voltige horses).Tail raised also differed but only if voltige horses were included (H = 11.95, p = 0.035) and to a lesser extent rolling (H = 10.66, p = 0.058). More dressage and high school horses than jumping horses (U = 484, p = 0.011 and U = 232, p = 0.0047 respectively) and advanced school horses (U = 218.5, p = 0.036; U = 104.5, p = 0.018 respectively) performed passage. Dressage horses raised their tails more than jumping (U = 368.5, p = 0.003) and voltige (U = 30, p = 0.027) horses and also cantered more than did eventing (U = 93.5, p = 0.038) and voltige (U = 31, p = 0.036) horses. Both dressage and high school horses showed more vigilance than jumping horses (U = 439.5, p = 0.008; U = 230, p = 0.024). Voltige horses rolled more frequently than did dressage (U = 25.5, p = 0.01), high school (U = 10.5, p = 0.036) or eventing (U = 9.5, p = 0.031) horses.

Success in the bridge test appeared influenced by type of work (Kruskal Wallis, H = 19.59, p = 0.0015; H = 14.16, p = 0.0068 without voltige): voltige (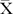 = 134.20±260.47 s) and jumping horses delayed less before crossing (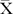 = 197.66±231.85 s) than the other horses: eventing: 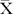 = 294.18±245.35 s; advanced school: 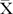 = 381.78±240.09 s; high School: 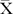 = 277.60±245.68 s; dressage: 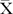 = 375.69±259.51 s) (Mann Whitney U test p<0.05 in all cases) (Figure S1 b). This was also true when only French saddlebreds were considered (N = 89, H = 12.9, p = 0.024).

## Discussion

Behavioural tests evaluating personality traits of adult horses revealed that type of work influenced their emotional level when facing a challenge. Despite having been accustomed to the test arena, some of the experienced horses could react strongly when released alone in the arena or when it included a novel object.

More than their overall emotional level (indices differed slightly), horses from different types of work differed in their interest in the object (voltige or jumping horses) or their tendency to perform more locomotion and excited behaviour such as passage, tail raised, vigilance, characteristic of high arousal / alarm levels [29], [30]. Voltige horses showed the quietest profiles (e.g. slow walk and rolling) when released and were less fearful when led over an unknown obstacle.

Slight differences were observed between the two breeds confirming both their genetic proximity [31] and differences revealed by the bridge test [19]. Differences observed according to type of work confirm earlier reports showing that dressage horses are overall emotionally more reactive [19], [32].

Dressage and high school horses showed similar behavioural tendencies, further confirming that work characteristics are implied, high school being a more elaborate form of dressage. Previous observations showed that frequency and type of stereotypic behaviour performed by adult dressage and high school horses in their box were similar (and high) [9]. Potential impact of type of work on the daily life of horses is thus further confirmed, as no other (genetic or environmental) factor could account for the differences observed. As manipulation of type of work was out of question, the possibility that stall managers unconsciously took behavioural characteristics into account when allocating horses to different types of work cannot be excluded. If this is the case, intrinsic characteristics and work particularities may well have additive effects that could explaining further some of the important differences observed.

The horses expressed similar behavioural profiles in the arena and the novel object test. Such similarities in the reactions to these two tests confirms other studies in adults [19], but not in younger horses [10]. This could be explained by the fact that these horses lived in single boxes with little social contact and were used working alone. They were therefore not reacting strongly to social separation (no whinnies, see also [31]), contrary to young horses living in groups or to riding-centre horses used working with others [16]. The reactions of our subjects were therefore related more probably to the strange (for them) situation of being released in the arena, a situation which never occurs otherwise, and being confronted with a novel object.

In these same unusual situations, our subjects reacted very differently and one axis of the FCA segregated slow walk and quieter behaviours from intense locomotor components and the other axis segregated gazing at objects from other behavioural patterns. Interestingly, Visser et al. [11] found the same two axes for young non-working Dutch warmbloods confronted with a novel object. The terms of “flightiness” and “sensitiveness” were proposed to describe these axes, which may well be reflecting general personality traits. Momozawa et al. [18] and Lloyd et al. [20] found that “anxiety” could be a reliable trait. Lloyd et al. [14] found a correlation between anxiousness (as assessed by questionnaires) and the frequency of “passage” performed during tests. According to this criterion, our dressage and high school horses were clearly more “anxious” than the other horses. Interestingly, both higher levels of “anxiety” and increased occurrence of stereotypies occur for the same type of work [9]. Increased anxiety is, according to O'Brien et al. [3], often found in cases of work stress. Remember that dressage/high school horses, because they are maintained strongly under control and their pace restrained, may experience at times conflicting relationships with their riders (e.g. through bit pressure [33]) and are not allowed to express any kind of emotion, a source of stress in humans [7]. On the other hand, the fact that dressage riders expect their horses to react quickly to their orders may develop their “sensitiveness” (in Visser et al.'s [11] sense) to the point that it can easily lead to nervousness, and by repetition and in the long term become an integral part of the horse's personality such as the “anxiousness” defined by Lloyd et al. [14]. Probably the more recent selection of bloodlines for dressage may increase even more this impact of type of work on behaviour [19]. Jumping and voltige horses have more chances to express locomotion needs at least, which may explain their quieter responses to the tests and in a handling / fear situation. This is to our knowledge the first evidence of a clear relationship between type of work and personality, in a context where type of work was the only factor that varied, potentially adding to intrinsic individual characteristics. Our results support reports suggesting that type of work may be an important factor in the development humans' personalities.

## Supporting Information

Figure S1 Emotionality in relation to type of work. Adv Sch = advanced school; Hi Sch = High School. a) Emotionality indices in relation to type of work (novel object test); Only trends were observed (Kruskal Wallis test: H = 3.41, P = 0.06); Means±standard error. b) Time required to cross the bridge in relation to type of work; Clear differences appeared between groups (Kruskal Wallis test: H = 19.6, P = 0.0015); Means±standard error; * p<0.05
(0.11 MB TIF)

Figure S2 FCA on frequencies of behaviours in the novel object test. Eventing, jumping, dressage, high school, advanced school = type of work. Trot passage, canter, vigilance, walk, slow walk, tail raised, rolling, pawing, snorting, touch, gaze = behaviours.
(0.40 MB TIF)

Figure S3 FCA on frequencies of behaviours in the arena test. Eventing, jumping, dressage, high school, advanced school = type of work. Trot, canter, vigilance, passage, tail raised, rolling, pawing, snorting = behaviours.
(0.46 MB TIF)

Appendix S1 Type of work
(0.03 MB DOC)
