# Supplementary figures and images for: Does Work Affect Personality? A Study in Horses
Source: PLoS One. 2011 Feb 9;6(2):e14659. doi: 10.1371/journal.pone.0014659 (PMC3036583; doi:10.1371/journal.pone.0014659)

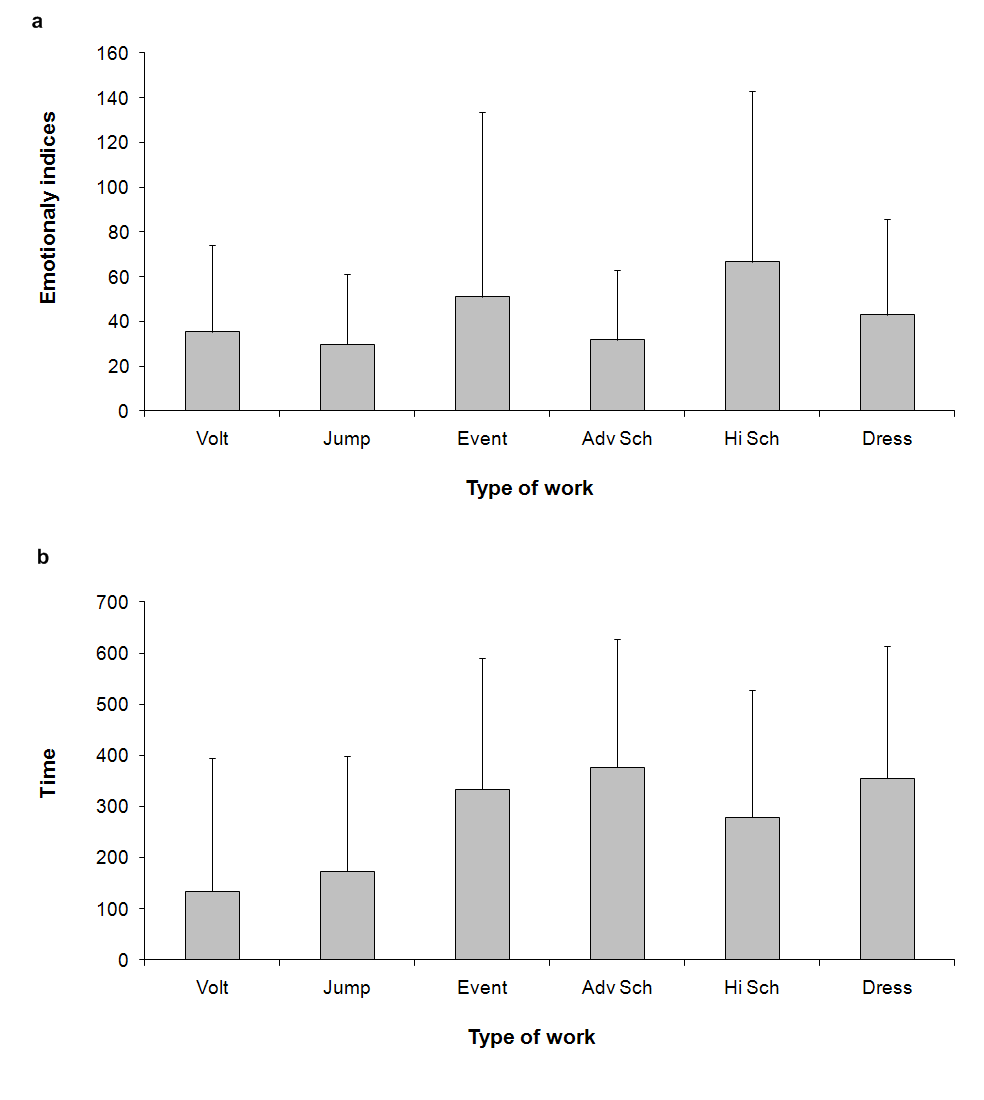

Supplement: Figure S1 — Emotionality in relation to type of work. Adv Sch = advanced school; Hi Sch = High School. a) Emotionality indices in relation to type of work (novel object test); Only trends were observed (Kruskal Wallis test: H = 3.41, P = 0.06); Means±standard error. b) Time required to cross the bridge in relation to type of work; Clear differences appeared between groups (Kruskal Wallis test: H = 19.6, P = 0.0015); Means±standard error; * p<0.05 (0.11 MB TIF) [file pone.0014659.s001.tif]

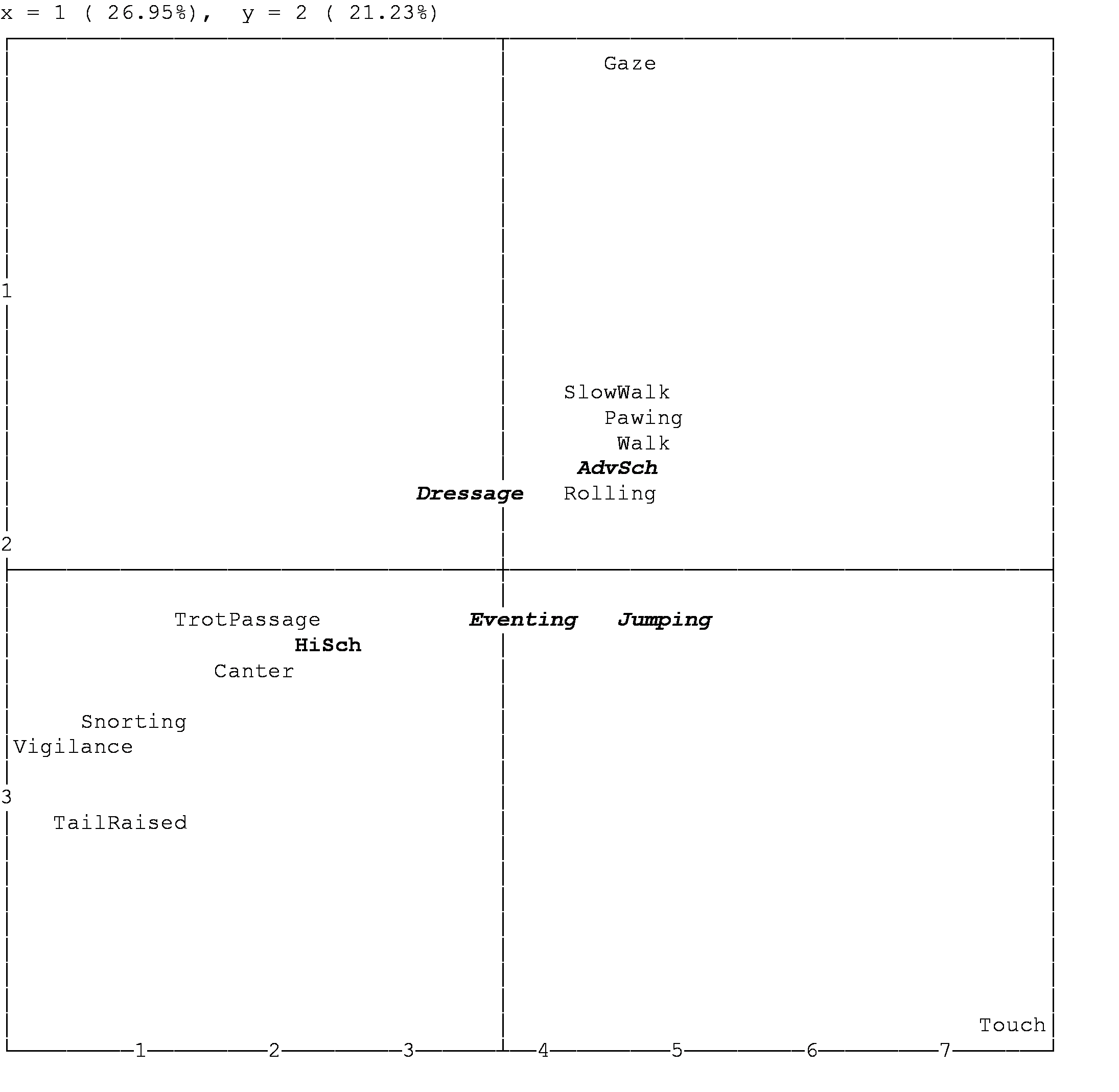

Supplement: Figure S2 — FCA on frequencies of behaviours in the novel object test. Eventing, jumping, dressage, high school, advanced school = type of work. Trot passage, canter, vigilance, walk, slow walk, tail raised, rolling, pawing, snorting, touch, gaze = behaviours. (0.40 MB TIF) [file pone.0014659.s002.tif]

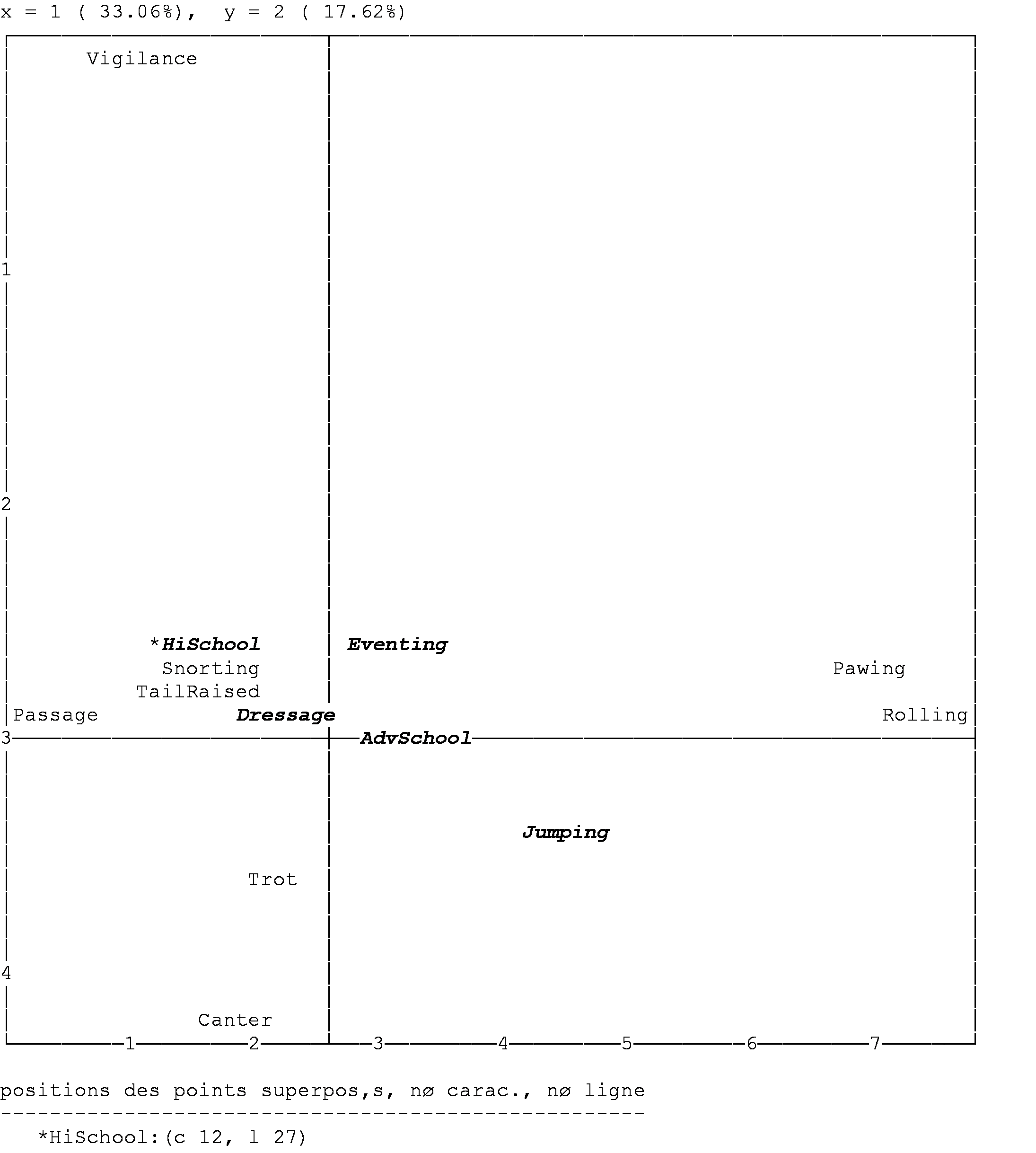

Supplement: Figure S3 — FCA on frequencies of behaviours in the arena test. Eventing, jumping, dressage, high school, advanced school = type of work. Trot, canter, vigilance, passage, tail raised, rolling, pawing, snorting = behaviours. (0.46 MB TIF) [file pone.0014659.s003.tif]
