# Supplementary material for: Does Work Affect Personality? A Study in Horses
Source: PLoS One. 2011 Feb 9;6(2):e14659. doi: 10.1371/journal.pone.0014659 (PMC3036583; doi:10.1371/journal.pone.0014659)
Supplement: Appendix S1 — Type of work (0.03 MB DOC) [file pone.0014659.s004.doc]

***Appendix S1***

Type of Work

**Show jumping**: horses are used to jump over a series of artificial obstacles in a limited time while being ridden by experienced riders.

**Dressage**: horses have to perform figures in an arena of a given area, such as circles, stops, and changing pace at given points in the arena, showing docility to riders' orders. Their paces are very controlled and neck is maintained flexed.

**High school riding**: horses perform the same tasks as in dressage but in addition must perform higher technical tasks and elevated paces (stamping, etc.).

**Eventing**: horses have to perform some dressage, jumping over artificial and natural obstacles while being ridden by experienced riders.

**Voltige:** horses have to turn in circles with their trainer in the middle using a long lunge. The horses have to maintain a regular pace (mostly slow canter) while riders jump onto their backs and may perform different exercises (e.g., standing on the horse's back). Orders are mainly given by voice.

**Advanced riding school**: horses were used to train experienced riders in order to prepare them to become instructors. They performed a variety of tasks, mostly jumping, eventing and training the riders’seats.
